# Supplementary material for: Drug repurposing for aging research using model organisms
Source: Aging Cell. 2017 Jun 16;16(5):1006–15. doi: 10.1111/acel.12626 (PMC5595691; doi:10.1111/acel.12626)
Supplement: Supplementary file 7 — Data S1 Zip‐Archive of all report cards. [file ACEL-16-1006-s007.zip › RC_OX5.pdf]

OX5

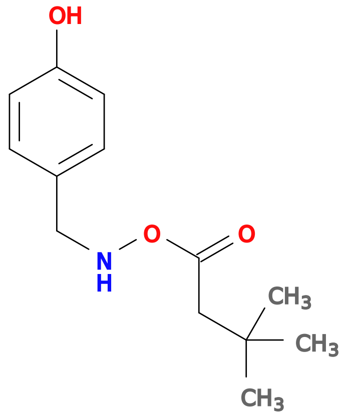

Database identifiers

ChEMBLCompound	CHEMBL256315
DrugBank	DB08335

Ranking

	Rank	Score
Drosophila	NA	NA
C. elegans	375/591	0.158

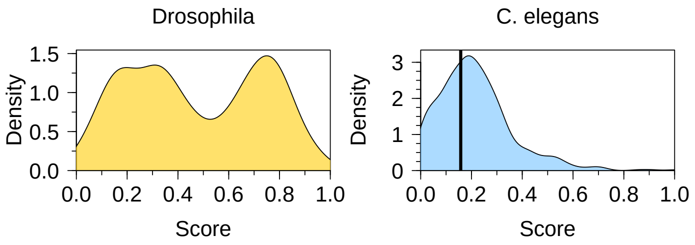

	Ageing implication	Domain conservation	Binding site conservation	Binding affinity	Bioavailability	Lipinski	Promiscuity	Purchasability	Drug approval	**Total**
Drosophila	NA	NA	NA	NA	NA	NA	NA	NA	NA	NA
C. elegans	0.81	0.855	0.887	0.461	0.294	0.0	-0.0	0.0	0.075	0.158

Names

- (2R,3R,4R,5R)-2-(hydroxymethyl)piperidine-3,4,5-triol
- (2R,3R,4R,5R)-2-methylolpiperidine-3,4,5-triol
- 1,5-dideoxy-1,5-imino-d-mannitol
- 2-hydroxymethyl-piperidine-3,4,5-triol
- 3,4,5-Piperidinetriol, 2-(hydroxymethyl)-, (2R,3R,4R,5R)-
- 4-HYDROXYBENZALDEHYDE O-(3,3-DIMETHYLBUTANOYL)OXIME
- BSPBio$_0$00991*Deoxymannojirimycin*

- DMJ
- Nsc625109 (hydrochloride)
- Ox5

Roles

ChEBI entry None has no roles

Status

Approved drug (according to ChEMBL)	No
Classification (according to DrugBank)	experimental
Number of Rule of 5 violations	0
Binding affinity to original target in log units (RF-Score prediction)	4.84
Burns *C. elegans* bioavailability prediction	-0.55

Compound Target Characteristics

Macrophage migration inhibitory factor

Best gene implication in ageing for this target family came from gene P34884 annotated in UniProt release 2014_02. Annotation GO subterm of 7568 (aging) was Inferred from Mutant Phenotype

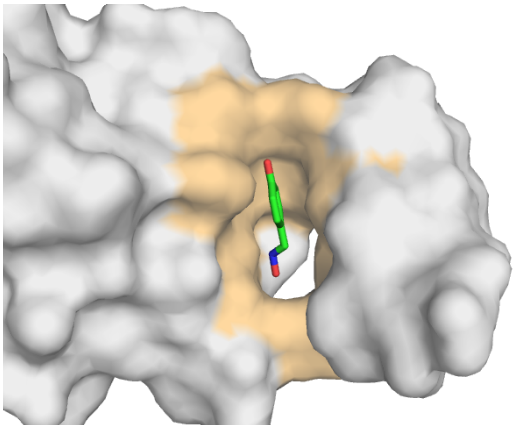

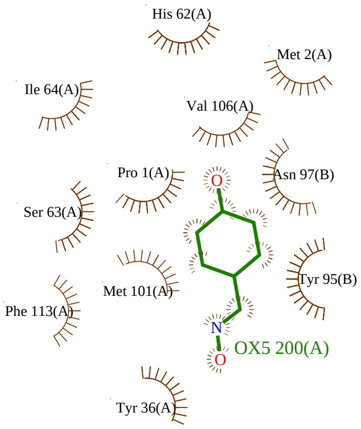

protein	amino acids contacts (binding site)
PDB:2ooz:chainA:P14174	P M Y H S I M V F
sp:P14174:MIF_HUMAN	P M Y H S I M V F
tr:I4AY87:I4AY87_HUMAN	P M Y H S I M V F
sp:P30904:MIF_RAT	P M Y H S I M V F
tr:D3ZE63:D3ZE63_RAT	P M Y H S I M V F
tr:D4A3P7:D4A3P7_RAT	P M Y H S I M V F
sp:P34884:MIF_MOUSE	P M Y H S I M V F
tr:Q545F0:Q545F0_MOUSE	P M Y H S I M V F
tr:Q9U228:Q9U228_CAEEL	P V Y K S I L I F
sp:Q18785:MIF2_CAEEL	P M R K S I L V V
tr:G3MU38:G3MU38_CAEEL	P M R K S I L V V

protein	whole protein ident	whole protein simil	domain-based ident	domain-based simil	contact-based ident	contact-based simil
PDB:2ooz:chainA:P14174	1.0	1.0	1.0	1.0	1.0	1.0
sp:P14174:MIF_HUMAN	1.0	1.0	1.0	1.0	1.0	1.0
tr:I4AY87:I4AY87_HUMAN	1.0	1.0	1.0	1.0	1.0	1.0
sp:P30904:MIF_RAT	0.9	0.98	0.9	0.98	1.0	1.0
tr:D3ZE63:D3ZE63_RAT	0.86	0.96	0.86	0.96	1.0	1.0
tr:D4A3P7:D4A3P7_RAT	0.83	0.96	0.83	0.96	1.0	1.0
sp:P34884:MIF_MOUSE	0.9	0.97	0.89	0.97	1.0	1.0
tr:Q545F0:Q545F0_MOUSE	0.9	0.97	0.89	0.97	1.0	1.0
tr:Q9U228:Q9U228_CAEEL	0.33	0.78	0.33	0.78	0.56	0.89
sp:Q18785:MIF2_CAEEL	0.32	0.73	0.32	0.76	0.56	0.8
tr:G3MU38:G3MU38_CAEEL	0.27	0.62	0.32	0.76	0.56	0.8
